# Aire controls the recirculation of murine Foxp3^+^ regulatory T‐cells back to the thymus

**DOI:** 10.1002/eji.201747375

**Published:** 2018-01-29

**Authors:** Jennifer E. Cowan, Song Baik, Nicholas I. McCarthy, Sonia M. Parnell, Andrea J. White, William E. Jenkinson, Graham Anderson

**Affiliations:** ^1^ Institute of Immunology and Immunotherapy College of Medical and Dental Sciences Medical School University of Birmingham Edgbaston Birmingham UK

**Keywords:** Thymic epithelium, Thymic selection, Thymocyte, Thymopoiesis, Thymus

## Abstract

In the thymus, medullary thymic epithelial cells (mTEC) determine the fate of newly selected CD4^+^ and CD8^+^ single positive (SP) thymocytes. For example, mTEC expression of Aire controls intrathymic self‐antigen availability for negative selection. Interestingly, alterations in both Foxp3^+^ Regulatory T‐cells (T‐Reg) and conventional SP thymocytes in *Aire^−/−^* mice suggest additional, yet poorly understood, roles for Aire during intrathymic T‐cell development. To examine this, we analysed thymocytes from *Aire* ^−/−^ mice using Rag2GFP and Foxp3 expression, and a recently described CD69/MHCI subset definition of post‐selection CD4^+^ conventional thymocytes. We show that while Aire is dispensable for de novo generation of conventional αβT‐cells, it plays a key role in controlling the intrathymic T‐Reg pool. Surprisingly, a decline in intrathymic T‐Reg in *Aire^−/−^* mice maps to a reduction in mature recirculating Rag2GFP^−^ T‐Reg that express CCR6 and re‐enter the thymus from the periphery. Furthermore, we show mTEC expression of the CCR6 ligand CCL20 is reduced in *Aire^−/−^* mice, and that CCR6 is required for T‐Reg recirculation back to the thymus. Collectively, our study re‐defines requirements for late stage intrathymic αβT‐cell development, and demonstrates that Aire controls a CCR6‐CCL20 axis that determines the developmental makeup of the intrathymic T‐Reg pool.

## Introduction

In the thymus, distinct stromal cell types support the generation of functionally competent αβT‐cells. For example, cortical thymic epithelial cells (cTEC) are specialised for positive selection, and form ‘thymic nurse cell’ complexes 1, 2 with DP thymocytes to enable secondary TCRα rearrangements and enhance opportunities for further maturation 3. Complementary to cortex specialisation are unique features of the thymus medulla that control later stages of thymocyte development. While dendritic cells in the medulla mediate negative selection 4, medullary thymic epithelial cells (mTEC) express MHCII and CD80/CD86 5, 6, Aire 7, 8, 9, 10, 11, and CCL21 12, 13 to control the selection, differentiation and migration of newly generated CD4^+^ and CD8^+^ single positive (SP) thymocytes prior to thymic exit. Thus, cTEC/mTEC specialisation is an important feature of intrathymic microenvironments that guide step‐wise T‐cell development.

In order to mediate negative selection, mTEC express Aire and Fezf2 for the intrathymic expression of self‐antigens that eliminate autoreactive thymocytes by apoptosis 14, 15, 16, 17, 18. Significantly, while mTEC also support lineage divergence in CD4^+^ thymocytes to create Foxp3^+^ Regulatory T‐cells (T‐Reg) 19, 20, their role in this process is not fully understood. For example, while the intrathymic generation of naturally diverse polyclonal Foxp3^+^ T‐Reg is dependent upon Aire in the neonate 21, the presence of peripheral recirculating T‐Reg in the adult thymus 22, 23, 24, 25, 26 makes it difficult to accurately quantitate de novo T‐Reg development at this later stage of life. Thus, the impact of Aire on the adult intrathymic T‐Reg pool is unclear. In addition, the medulla has also been linked to stages in the post‐selection development of conventional SP thymocytes 27, 28. Importantly however, while CCR4, CCR7, CCR9 19, 29, 30 and CD69, 6C10, Qa2 31 define CD4^+^ SP thymocyte heterogeneity, recent work 28 has raised doubts about the suitability of some of these markers (e.g. Qa2) to identify specific developmental stages. Interestingly, the same study described ‘Semi‐Mature’ (SM, CD69^+^MHCI^lo^), ‘Mature 1’ (M1, CD69^+^MHCI^+^) and ‘Mature 2’ (M2, CD69^−^MHCI^+^) subsets that represent an accurate developmental sequence of CD4^+^ SP thymocytes, with functional maturation residing within M1 and M2 cells 28, 32. However, while these studies define SP thymocyte heterogeneity, the role of the thymus medulla in controlling transit through these stages is not clear. For example, while the reduction in late‐stage conventional CD4^+^ thymocytes in adult *Aire^−/−^* mice suggests a role for mTEC and Aire in post‐selection conventional thymocyte differentiation 31, 33, 34, other studies showed that mTEC are not required for conventional CD4^+^ thymocyte development 19. Consequently, the role of mTEC and Aire in late stage CD4^+^ αβT‐cell development is poorly understood.

Given these uncertainties, we have studied mechanisms controlling the developmental progression of CD4^+^ thymocytes, and examined the impact of the thymus medulla on this process. By analysing Rag2GFP expression in intrathymic Foxp3^+^ T‐Reg and SM/M1/M2 CD4^+^ SP conventional thymocyte subsets in adult *Aire^−/−^* mice, we show that Aire is dispensable for de novo conventional and Foxp3^+^ T‐Reg development. In contrast, we find that Aire exerts a surprising influence on the intrathymic T‐Reg pool, by controlling recirculation of peripheral CCR6^+^ T‐Reg back to the thymus. Moreover, we show this involves Aire‐mediated regulation of the chemokine CCL20 in mTEC, and identify a novel CCR6‐CCL20 axis for T‐Reg thymus recirculation. Collectively, our study redefines the requirements of post‐selection intrathymic T‐cell development, and describes a new role for Aire in controlling migration of T‐Reg between peripheral tissues and the thymus.

## Results

### Aire controls developmental heterogeneity in the intrathymic Foxp3^+^ T‐Reg pool

**Figure 1:**
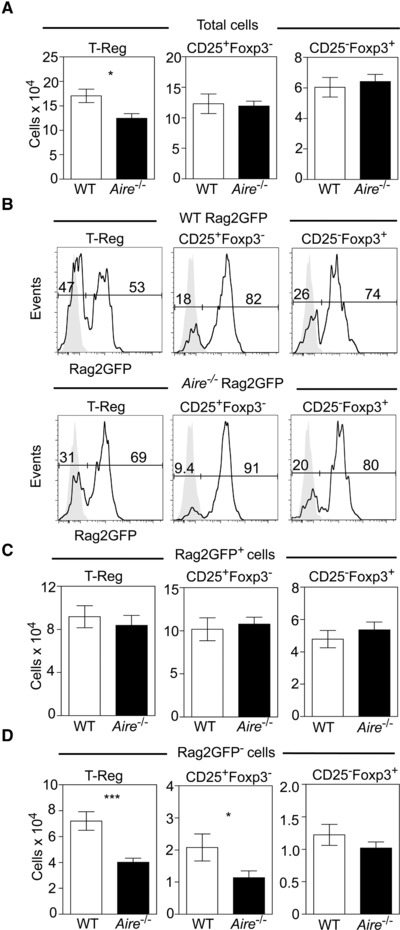
Selective reduction in Rag2GFP^−^ Foxp3^+^ thymic T‐Reg in *Aire^−/−^* mice. Flow cytometric analysis of CD4^+^CD8^−^TCRβ^+^CD25^+^Foxp3^+^, CD4^+^CD8^−^TCRβ^+^CD25^+^Foxp3^−^ and CD4^+^CD8^−^TCRβ^+^CD25^−^Foxp3^+^ thymocytes from both WTRag2GFP (white) and *Aire^−/−^*Rag2GFP (black) mice. Gating strategy is shown in Supporting Information Fig. 1. Bar graphs in panel (A) show total numbers of each population, and panel (B) shows representative examples of Rag2GFP expression within each population, gray histograms indicated fluorescence levels on GFP^−^ thymocytes. Panel (C) shows numbers of Rag2GFP^+^ T‐Reg, CD25^+^Foxp3^−^ and CD25^−^Foxp3^+^ cells in WTRag2GFP (white) and *Aire^−/−^*Rag2GFP (black) mice. Panel (D) shows numbers of Rag2GFP^−^ T‐Reg, CD25^+^Foxp3^−^ and CD25^−^Foxp3^+^ cells in WTRag2GFP (white) and *Aire^−/−^*Rag2GFP (black) mice. Error bars indicate SEM, using an unpaired Student's two‐tailed *t*‐test: *p < 0.05; ***p < 0.001. Data are pooled from at least three separate experiments, with at least 6 mice of each strain in total.

The thymus medulla contains Foxp3^+^ T‐Reg that branch off from the programme of conventional thymocyte development during the CD4^+^ stage 19, 35, 36, and mTEC play an essential role in the generation of Foxp3^+^ T‐Reg and their precursors 19, 20. Consistent with this, Aire influences Foxp3^+^ T‐Reg generation in neonatal mice 21. However, as the naturally diverse T‐Reg pool in the adult thymus consists of both newly produced cells and recirculating T‐Reg that have re‐entered from the periphery 22, 23, 24, the requirement for Aire during adult intrathymic T‐Reg development is not clear. To address this directly, we crossed *Aire^−/−^* mice with Rag2GFP mice, providing an accurate means to distinguish newly produced (GFP^+^) and recirculating (GFP^−^) cells in the thymus 22, 23, and examined the developmental makeup of thymic T‐Reg. The gating strategy used to define intrathymic Foxp3^+^ T‐Reg development is shown in Supporting Information Fig. 1. Interestingly, while total T‐Reg numbers showed a significant reduction in *Aire^−/−^*Rag2GFP mice (Fig. 1A), the numbers of CD25^+^Foxp3^−^ and CD25^−^Foxp3^+^ thymocytes that represent T‐Reg progenitors 35, 36 were unchanged (Fig. 1A). Moreover, when we analysed GFP expression in both T‐Reg and T‐Reg progenitor populations from thymuses of WTRag2GFP and *Aire^−/−^*Rag2GFP mice, we saw no differences in the absolute numbers of GFP^+^ thymocytes representing de novo generated cells (Fig. 1B and C). Thus, newly generated Rag2GFP^+^ T‐Reg and T‐Reg progenitors develop effectively in the absence of Aire, indicating that the reduction in total T‐Reg in *Aire^−/−^* mice cannot be explained by a role for Aire in intrathymic T‐Reg development. Surprisingly, further analysis of *Aire^−/−^*Rag2GFP mice revealed a significant decrease in the number of intrathymic T‐Reg that lacked GFP expression (Fig. 1D), indicating that a selective decline in Rag2GFP^−^ T‐Reg provides an explanation for the reduction in overall intrathymic T‐Reg numbers in *Aire^−/−^* mice. As these cells have been shown previously to represent thymus‐recirculating cells 22, 23, this suggests that Aire influences the thymus re‐entry of mature T‐Reg from the periphery.

### Aire controls recirculation of peripheral T‐Reg back to the thymus

**Figure 2:**
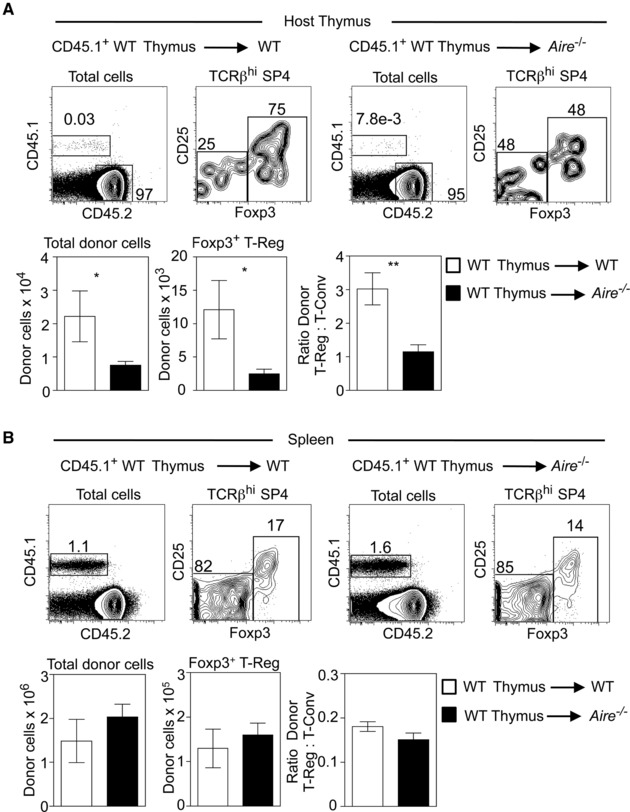
Aire regulates the thymus recirculation of peripheral Foxp3^+^ T‐Reg. panel (A) shows flow cytometric analysis of the host thymus of either CD45.2^+^ WT or *Aire^−/−^* mice, previously grafted with an embryonic CD45.1^+^ WT thymus. Upper panels show representative examples of the detection of graft‐derived CD45.1^+^CD45.2^−^ thymic cells in the host mouse thymus, and detection of CD25^+^Foxp3^+^ T‐Reg within these cells. Lower panels show absolute cell numbers of graft‐derived donor cells, and the ratio of donor T‐Reg:T‐conv present in the thymus of WT (white bars) and *Aire^−/−^* (black bars) mice. Panel (B) shows identical analysis of splenocytes from WT (white bars) and *Aire^−/−^* (black bars) mice transplanted with a CD45.1^+^ WT embryonic thymus. Error bars indicate SEM, using an unpaired Student's two‐tailed *t*‐test: *p < 0.05; **p < 0.01. Data are representative of at least two separate experiments, with at least 5 mice of each strain in total.

To directly examine the requirement for Aire in thymus T‐Reg recirculation, we performed thymus transplantation experiments to visualise and quantitate peripheral T‐Reg homing to the thymus. Thus, freshly isolated WT embryonic day 18 (E18) thymus lobes from BoyJ mice, which contain a cohort of CD45.1^+^ congenically marked thymocytes, were transplanted under the kidney capsule of CD45.2^+^ WT or CD45.2^+^ *Aire^−/−^* adult mice. In this way, grafted thymuses generate a single wave of T‐cell development, and so allow direct analysis of thymus recirculation through the identification of graft‐derived CD45.1^+^ T‐cells within the host thymus 22. After 5 weeks, host thymus and spleen tissues were harvested from WT and *Aire^−/−^* hosts, and CD45.1 expression was used to identify graft‐derived cells alongside analysis of CD4, CD8, TCRβ and Foxp3 by flow cytometry. When we analysed host thymuses from WT and *Aire^−/−^* mice, we saw striking differences in the frequency of graft‐derived T‐cells. Thus, the numbers of both total donor T‐cells and Foxp3^+^ T‐Reg, were significantly reduced in the *Aire^−/−^* host thymus (Fig. 2A). Moreover, while in the WT host thymus, graft‐derived T‐Reg:T‐conv were present at a ratio of 3:1, in the thymus of *Aire^−/−^* hosts we saw a markedly skewed ratio of 1:1, indicating reduced graft‐derived T‐Reg entry to the *Aire^−/−^* thymus (Fig. 2A). Importantly, in the spleens of both WT and *Aire^−/−^* mice, the numbers of total graft‐derived CD45.1^+^ donor cells and CD45.1^+^Foxp3^+^ T‐Reg, were comparable (Fig. 2B). Moreover, splenic ratios of T‐Reg:T‐conv CD45.1^+^ graft‐derived T‐cells were comparable in both WT and *Aire^−/−^* mice (Fig. 2B). Thus, WT thymus‐graft derived T‐Reg are represented equally in the spleen of WT and *Aire^−/−^* hosts, indicating that limitations in their availability cannot explain the reduction in thymus‐recirculating cells in *Aire^−/−^* mice. Rather, these findings indicate that absence of Aire limits the entry of peripheral T‐Reg to thymus.

### Aire controls a CCL20‐CCR6 axis for thymus T‐Reg recirculation

**Figure 3:**
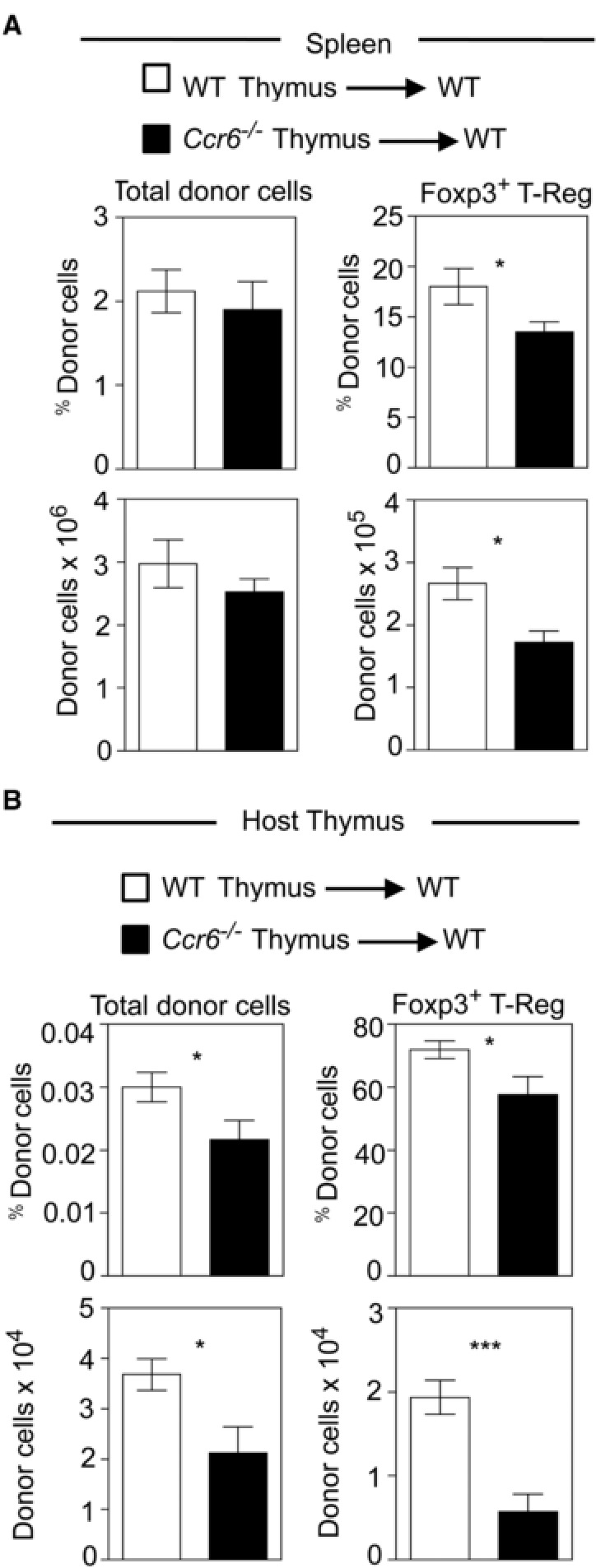
CCR6 controls the thymus homing of peripheral Foxp3^+^ T‐Reg. panel (A) shows flow cytometry analysis of splenocytes obtained from CD45.1^+^ WT mice previously grafted with either CD45.2^+^ WT (white bars) or *Ccr6^−/−^* (black bars) embryonic thymus lobes. Graphs show the absolute numbers of indicated donor cell populations. Panel (B) shows similar analysis of graft‐derived donor cells in the host thymus harvested from the same mice. Error bars indicate SEM using an unpaired Student's two‐tailed *t*‐test: ^*^ *p* < 0.05; ^***^ *p* < 0.001. Data are representative of at least two separate experiments, with at least 5 mice of each strain in total.

Within the intrathymic T‐Reg pool, recirculating T‐Reg can be distinguished from newly generated T‐Reg by their differential expression of chemokine receptors. Thus, while newly produced Rag2GFP^+^ T‐Reg contain CCR6^−^CCR7^+^ cells, recirculating Rag2GFP^−^ cells are CCR6^+^CCR7^−^ 22. As CCR6 is expressed by peripherally‐derived T‐Reg within the intrathymic pool, we next examined the involvement of this chemokine receptor in thymic recirculation. Initially, we transplanted lymphoid CD45.2^+^ embryonic thymus lobes from either WT or *Ccr6^−/−^* mice into congenically marked CD45.1^+^ WT mice. After 5 weeks, thymus and spleen from host mice was harvested, and the frequency and phenotype of graft‐derived CD45.2^+^ T‐cells was determined by flow cytometry. In the spleen, donor‐derived Foxp3^+^ T‐Reg numbers from *Ccr6^−/−^* thymus grafts were only slightly lower than those from WT grafts (Fig. 3A). However, when we analysed the number of graft‐derived cells in the host thymus, we saw a far greater reduction in *Ccr6^−/−^* graft‐derived T‐Reg compared to WT graft‐derived cells (Fig. 3B).

**Figure 4:**
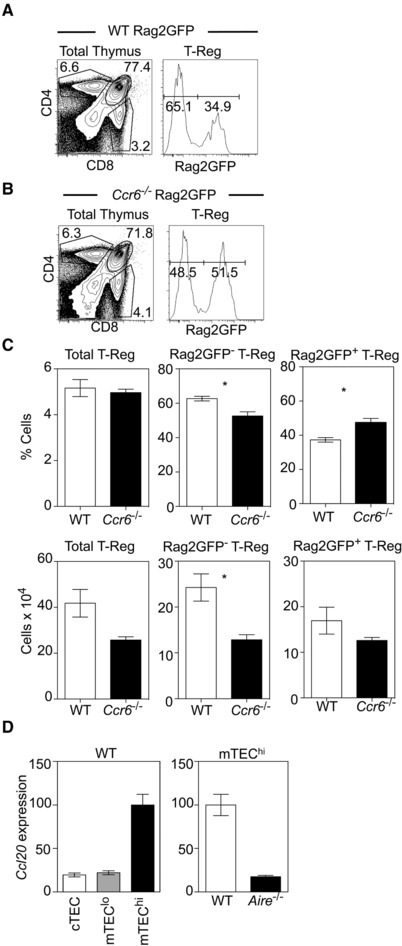
Recirculating T‐Reg are reduced in the thymus of *Ccr6* ^−/−^Rag2GFP mice. Flow cytometry analysis of CD4, CD8 expression in WTRag2GFP (Panel A) and *Ccr6^−/−^*Rag2GFP (Panel B) thymocytes, together with Rag2GFP levels on CD25^+^Foxp3^+^ SP4 thymic T‐Reg. Panel (C) shows percentages (upper panels) and absolute numbers (lower panels) of Rag2GFP^+^ and Rag2GFP^−^ thymic T‐Reg in WTRag2GFP (white bars) and Ccr6^−/−^Rag2GFP (black bars) mice. Error bars indicate SEM using an unpaired Student's two‐tailed *t*‐test: ^*^ *p* < 0.05. Data are pooled from at least three separate experiments, with at least 6 mice of each strain in total. Panel (D) shows qPCR analysis of *Ccl20* mRNA levels in indicated TEC populations isolated from WT mice, and in mTEC^hi^ isolated from either WT or *Aire^−/−^* mice. Fold levels represent the mean +/**‐**SEM of replicate reactions. Data is representative of at least 2 independent experiments, where cDNA samples were prepared from sorted cells from at least 3 mice.

As the above findings suggest that entry of peripheral *Ccr6^−/−^* T‐Reg into the host thymus is impaired, and to further examine the role of CCR6 in thymus T‐Reg recirculation, we next generated *Ccr6^−/−^*Rag2GFP mice and analysed developmental heterogeneity within the intrathymic T‐Reg pool (Fig. 4A and B). Importantly, by discriminating between recirculating GFP^−^ cells and newly produced GFP^+^ cells, we saw a significant reduction in both the percentage and number of Rag2GFP^−^ T‐Reg in the thymus of *Ccr6^−/−^*Rag2GFP mice (Fig. 4C). Thus, by both thymus transplantation and Rag2GFP expression analysis, these findings suggest that CCR6 is involved in the migration of peripheral T‐Reg back to the thymus. Finally, we next examined how CCR6 may be linked to the requirement for Aire in this process. As Aire has been shown to influence expression of intrathymic chemokines 33, 37, 38, 39, we analysed expression of the CCR6 ligand, CCL20, in purified TEC populations isolated from WT and *Aire^−/−^* mice. Interestingly, we found that *Ccl20* mRNA was selectively expressed by mTEC^hi^ (Fig. 4D), the subset that contains Aire‐expressing cells. Moreover, direct comparison of mTEC^hi^ from WT and *Aire^−/−^* mice showed a striking reduction of *Ccl20* mRNA in the latter (Fig. 4D). Thus, mTEC^hi^ express CCL20, which is reduced in the absence of Aire. Taken together with the expression of CCR6 by recirculating T‐Reg, and the diminished thymus recirculation of *Ccr6^−/−^* T‐Reg, our data suggest that Aire regulates a CCL20‐CCR6 axis required for peripheral T‐Reg homing to the thymus.

### Aire is not required for conventional CD4^+^ thymocyte development

**Figure 5:**
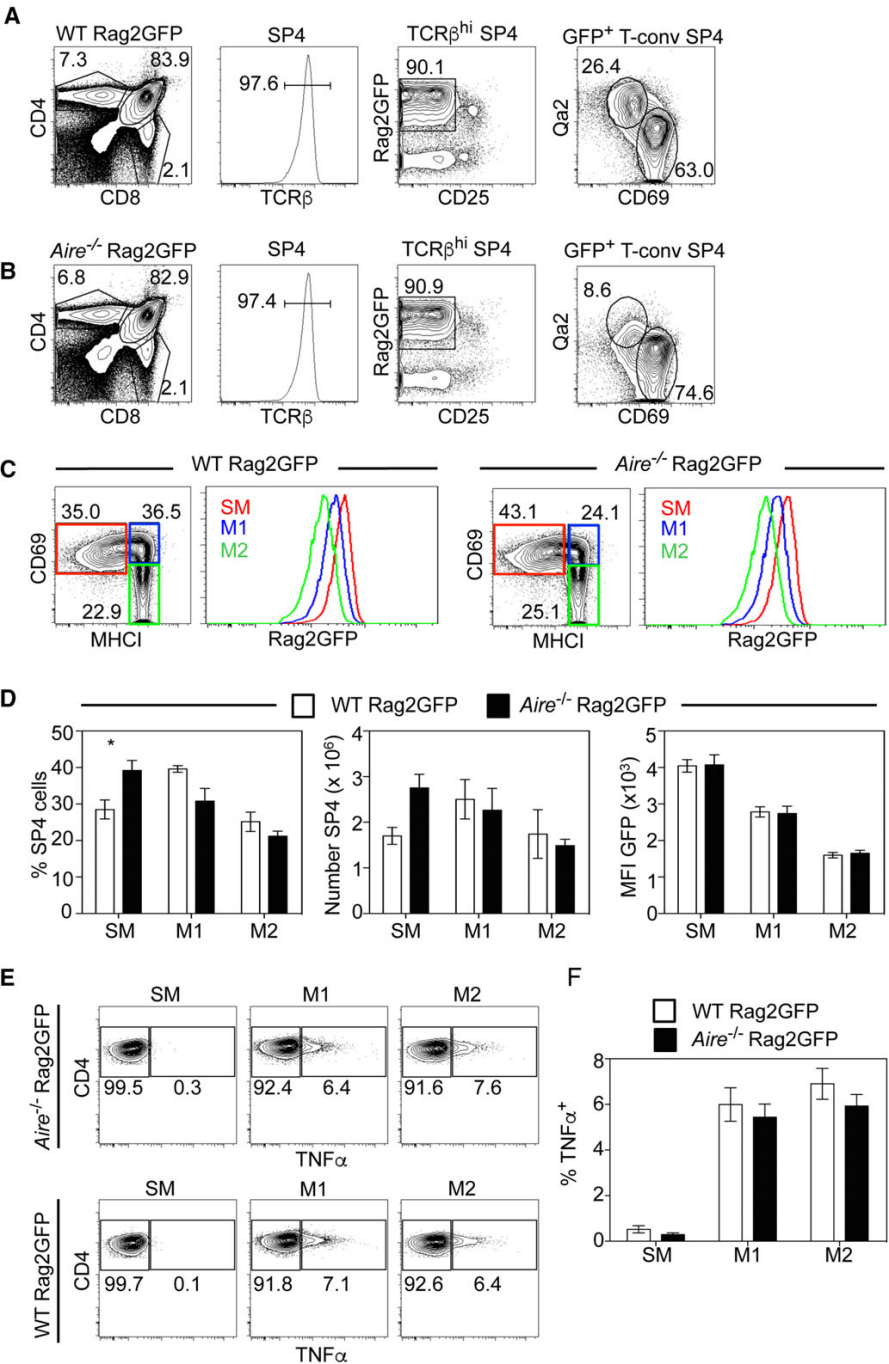
Aire is dispensable for post‐selection maturation and cytokine licencing in conventional thymocytes. Flow cytometric detection of CD4^+^CD8^−^TCRβ^+^CD25^−^Rag2GFP^+^ conventional CD4^+^ SP thymocytes in WTRag2GFP (A) and *Aire^−/−^*Rag2GFP (B) mice. Analysis of Qa2/CD69 expression in these cells is also shown. Panel (C) shows conventional CD4^+^ SP thymocytes from WTRag2GFP and *Aire^−/−^*Rag2GFP mice, analysed for expression of CD69 and MHCI to detect: SM (CD69^+^MHCI^low^), M1 (CD69^+^MHCI^+^) and M2 (CD69^−^MHCI^+^) subsets. Histograms show the Rag2GFP levels in the indicated populations. Panel (D) shows proportions, numbers and Rag2GFP levels of populations identified in (C). Panels (E) and (F) show analysis of TNFα production in SM, M1 and M2 subsets of conventional CD4^+^ SP thymocytes from WTRag2GFP (white bars) and *Aire^−/−^*Rag2GFP (black bars) mice. Error bars indicate SEM, and a one‐way ANOVA was used, ^*^ *p*< 0.05. Data are pooled from at least three separate experiments, with at least 6 mice of each strain in total.

In addition to housing conventional Foxp3^+^ T‐Reg, the thymus medulla contains conventional CD4^+^ and CD8^+^ thymocytes that consist of a series of subsets that are phenotypically and developmentally distinct 19, 27, 28. While the mechanisms controlling conventional post‐selection thymocyte development are not fully understood, a reduction in Qa2^+^CD4^+^ thymocytes in *Aire* ^−/−^ mice suggests a role for Aire in this process 31. However, as recent studies have raised doubts about the suitability of some previously used markers as indicators of maturation status 28, we adopted a method involving Rag2GFP, MHCI and CD69 expression that robustly identifies a developmental programme for CD4^+^ thymocytes 28. To directly assess the role of Aire in conventional thymocyte development, we analysed *Aire^−/−^*Rag2GFP mice in relation to semi‐mature (SM, CD69^+^MHCI^lo^), mature 1 (M1, CD69^+^MHCI^hi^) and mature 2 (M2, CD69^+^MHCI^hi^) CD4^+^ thymocyte subsets. In all cases, conventional CD4^+^ thymocytes were identified as CD4^+^8^−^TCRβ^+^Rag2GFP^+^CD25^−^ cells (Fig. 5A and B). As shown previously 31, analysis of CD69 and Qa2 expression showed a marked reduction in Qa2^hi^CD69^−^ cells in *Aire^−/−^*Rag2GFP mice, reported to represent the most mature CD4^+^ cells (Fig. 5A and B). In contrast, SM, M1 and M2 CD4^+^ subsets defined by MHCI, CD69 and Rag2GFP were readily detectable in both WTRag2GFP and *Aire^−/−^*Rag2GFP mice, which also showed a comparable progressive reduction in Rag2GFP levels (Fig. 5C and D). Moreover, while the percentage of SM cells in *Aire^−/−^*Rag2GFP mice showed a slight increase, no significant differences in the absolute numbers of SM, M1 and M2 CD4^+^ cells from WTRag2GFP and *Aire^−/−^* Rag2GFP mice were seen (Fig. 5D). Thus, our analysis of *Aire^−/−^* mice using Rag2GFP as a reporter of developmental status in association with SM/M1/M2 subset detection indicates that Aire is not required for conventional CD4^+^ thymocyte development. Moreover, that an unperturbed programme of maturation in *Aire^−/−^* mice is still accompanied by alterations in Qa2^+^ thymocyte frequency confirms the limited suitability of this marker to examine post‐selection thymocyte differentiation 28.

In addition to phenotypic changes, CD4^+^ thymocytes undergo functional maturation as they progress through post‐selection stages of development 27, 28, 32, 40. This includes the ability to produce the cytokine TNFα in response to TCR stimulation, a process termed cytokine licencing 28. To examine whether Aire plays a role in the functional maturation of CD4^+^ thymocytes, we next assessed the cytokine licencing capabilities of SM, M1 and M2 subsets from WT and *Aire* ^−/−^ mice. Thymocytes were stimulated with anti‐CD3/anti‐CD28 and TNFα production was analysed following cell permeabilisation 28. Interestingly, CD4^+^ thymocytes from both WTRag2GFP and *Aire^−/−^*Rag2GFP mice were comparable in their TNFα production (Fig. 5E and F). Thus, consistent with their immature status, stimulation of the least mature SM CD4^+^ cells from both WTRag2GFP and *Aire^−/−^*Rag2GFP mice failed to induce TNFα expression. However, more mature M1 and M2 subsets from both WTRag2GFP and *Aire^−/−^*Rag2GFP mice generated TNFα‐producing cells at similar frequencies (Fig. 5E and F). Thus, the ability of CD4^+^ thymocytes to acquire cytokine licencing properties, and the developmental timing of this process, occurs normally in *Aire^−/−^* mice. Taken together with our phenotypic analysis of post‐selection maturation stages, these findings demonstrate that Aire is not required for completion of intrathymic CD4^+^ thymocyte development.

## Discussion

The thymus medulla houses both conventional and Foxp3^+^ T‐Reg generated during T‐cell development 41, 42. As a consequence, medullary functions are diverse in order to influence multiple events within distinct αβT‐cell lineages. The importance of the medulla is well studied, which is due at least in part to mTEC expression of Aire, and its control of intrathymic availability of self‐antigens for negative selection 11, 15, 16. Interestingly, Aire has been implicated in other aspects of medulla function, including post‐selection development of conventional CD4^+^ thymocytes and Foxp3^+^ T‐Reg development 21, 31, 33. However, understanding these additional roles for Aire has been limited by uncertainty in identifying post‐selection stages in T‐cell development 28, and the presence of recirculating peripheral T‐Reg in the adult thymus, that hinder accurate analysis of de novo T‐Reg selection 22, 23, 24, 25. Here, we used a variety of approaches to examine mechanisms regulating CD4^+^ conventional and Foxp3^+^ T‐Reg in the thymus. For conventional thymocytes, we adopted an approach in which step‐wise progression through three SM, M1 and M2 CD4^+^ stages was examined alongside de novo Rag2GFP^+^ thymocyte maturation and the timing of acquisition of functional competency 28. By performing this analysis in *Aire^−/−^* mice, we show that SM/M1/M2 post‐selection phenotypic progression, and acquisition of TNFα cytokine licencing, both occur independently of Aire. Thus, Aire is not a key regulator of post‐selection thymocyte differentiation. This conclusion differs from earlier studies where a reduction in Qa2^hi^CD69^−^ CD4^+^ thymocytes in *Aire^−/−^* mice (also shown here) was taken as evidence for a role for Aire in post‐selection thymocyte maturation 31. Importantly, as Qa2 expression has recently been shown to indicate type 1 interferon signalling in thymocytes rather than their maturation status 28, its suitability as a marker to identify stages in post‐thymic differentiation is uncertain. Indeed, our finding that post‐selection thymocyte maturation is regulated by Aire‐independent mechanisms is compatible with studies showing that mTEC, major expressers of Aire in the thymus, are not required for the generation of mature CD4^+^ thymocytes 19. As such, our data suggest the primary role of Aire in conventional adult αβT‐cell development is tolerance induction, and not post‐selection differentiation.

**Figure 6:**
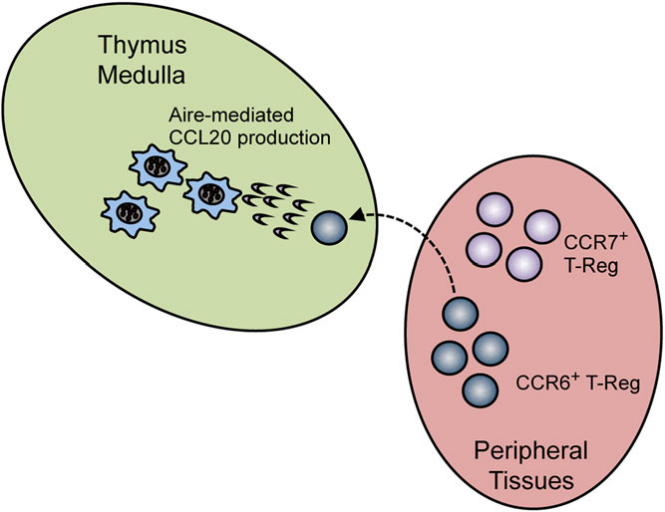
Aire controls A CCR6‐CCL20 axis for Foxp3^+^ T‐Reg recirculation to the thymus. In the model shown, mTEC produce the chemokine CCL20 in an Aire‐dependent manner. This enables mature CCR6^+^ T‐Reg to recirculate back to the thymus from peripheral tissues. Newly produced T‐Reg within peripheral tissues are CCR7^+^CCR6^−^, and so do not undergo CCL20‐mediated thymus recirculation.

Foxp3^+^ T‐Reg require the medulla, and mTEC in particular, for their development 19. Here, using Rag2GFP expression and thymus transplantation approaches, we reveal an unexpected impact of Aire on the developmental makeup of the intrathymic T‐Reg pool in adult mice, and show it controls the frequency of mature T‐Reg entering the thymus from the periphery. Thus, a reduction in T‐Reg recirculation, not de novo production, may explain the smaller T‐Reg pool size in the thymus of adult *Aire^−/−^* mice. In addition, it has also been shown that T‐Reg can be retained within the thymus for longer periods compared to conventional thymocytes 43. While these findings emphasise the developmental heterogeneity that exists within T‐Reg in the thymus, it is currently unclear whether Aire impacts the intrathymic T‐Reg pool by influencing both intrathymic retention and re‐entry from peripheral tissues. Importantly, these findings on the role of Aire in T‐Reg recirculation may still be consistent with studies demonstrating a requirement for Aire in TCR‐transgenic T‐Reg development in adult mice 20, 44, 45, 46. Thus, while we show naturally diverse intrathymic T‐Reg are produced in comparable numbers in WT and *Aire^−/−^* mice, they may differ in the αβTCR repertoires they express. This would be consistent with Aire controlling the development of those cells selected by Aire‐dependent TRAs. In addition, by demonstrating Aire‐dependent expression of CCL20 for entry of CCR6^+^ peripheral T‐Reg, we provide new insight into Aire's ability to exert influence on thymus populations by affecting intrathymic chemokine availability. Thus, while Aire controls XCL1‐mediated thymic DC positioning 37 and CCR4/CCR7 ligand‐mediated thymocyte migration 33, 38, we now show that Aire also controls CCL20 for T‐Reg migration between the thymus and periphery (Fig. 6). That CCR6 is expressed by recirculating cells within intrathymic T‐Reg 22 is consistent with this, and further highlights Aire's impact on T‐Reg extends beyond any influence on their intrathymic production. It is interesting to note that while thymic T‐Reg recirculation is reduced in both *Aire^−/−^* and *Ccr6^−/−^* mice, some peripherally‐derived thymic T‐Reg are still detectable. Whether this reflects additional roles for other Aire‐dependent and –independent chemokines is unclear.

The functional relevance of T‐Reg recirculation to the thymus is not fully understood. However, recent studies have shown that it may control de novo T‐Reg development by creating intrathymic competition for IL‐2 23, 24. However, despite reduced recirculating T‐Reg in *Aire^−/−^* mice, we saw no changes in the numbers of de novo produced T‐Reg precursors or more mature Foxp3^+^ T‐Reg. Whether the reduced peripheral T‐Reg numbers in the thymus of *Aire^−/−^* mice are still able to effectively compete for IL‐2 and limit new T‐Reg production is not clear. Interestingly, CCL20 directs CCR6^+^ T‐Reg to peripheral inflammatory sites 47, and its expression by mTEC for the attraction of CCR6^+^ T‐Reg correlates with conditions within medullary thymic microenvironments that support constitutive MHC class II expression and elevated co‐stimulatory molecules on thymic APC 48. Whether recirculating T‐Reg influence the thymic medullary microenvironments they reside within is currently not known.

In conclusion, by analysing conventional and Foxp3^+^ thymocytes and separating intrathymic production from thymic recirculation, we have examined the requirement for Aire in specialisation of the thymus medulla during αβT‐cell development. Collectively, our findings support models in which the major role of Aire^+^ medullary microenvironments in adult thymus is the control of tolerance induction and not thymocyte differentiation. In contrast, by revealing an Aire‐mediated mechanism that controls the entry of peripherally derived T‐Reg to the thymus, we demonstrate the ability of Aire to regulate communication between sites of T‐cell production and function.

## Materials and methods

### Mice

Wildtype (WT) C57BL/6, Rag2GFP 49, *Aire^−/−^* 50 and *Ccr6* ^−/−^ mice (obtained from The Jackson Laboratory, stock number 005793 51 on a C57BL/6 background were used at 8–12 weeks of age. All mice were housed at the Biomedical Services Unit at The University of Birmingham, and all procedures were performed with permission for the UK Home Office. Rag2GFP mice were interbred with *Aire^−/−^* and *Ccr6^−/−^* mice to generate *Aire* ^−/−^Rag2GFP and *Ccr6* ^−/−^Rag2GFP mice respectively. Embryos from WT CD45.1^+^ BoyJ and C57BL/6 CD45.2^+^ mice were generated from timed matings, with the day of vaginal plug detection designated as day 0.

### Thymocyte cytokine licencing

TNFα production in freshly isolated thymocytes was performed as described 28. Briefly, 5 × 10^6^ thymocytes were cultured with plate‐bound anti‐CD3 (10 ug/ml, clone 145‐2C11, eBioscience) and anti‐CD28 (20 ug/ml, clone 37.51, eBioscience) for 6 hours in GolgiPlug (BD Bioscience). Cells were stained with antibodies described below to detect SM, M1 and M2 CD4^+^ cells, fixed using BD Cytofix/Cytoperm (BD Bioscience) for 30 min on ice, and TNFα production was detected by flow cytometry using an anti‐TNFα antibody (MP6‐XT22, BD Bioscience).

### Antibodies, immunoconjugates and flow cytometry

Thymus and spleen were mechanically teased with glass slides to acquire single cell suspensions and cells were stained with antibodies to the following: CD45.1 (A20, eBioscience), CD45.2 (104, eBioscience), CD4 (RM4‐5, Biolegend), CD8 (53‐6.7, Biolegend), CD25 (PC61.5, Biolegend), CD44 (IM7, eBioscience), Qa2 (695H1‐9‐9, Biolegend), TCRβ (H57‐597, eBioscience), CD69 (H1.2F3, eBioscience), and H‐2K^b^ (AF6‐88.5, Biolegend). Reagents were conjugated to Pacific Blue, Brilliant Violet (BV) 421, BV510, BV711, PE, PE‐Cy7, PerCP–eFluor 710, allophycocyanin– eFluor 780 and Alexa Fluor 700. Streptavidin PE‐Cy7 (eBioscience) was used to detect biotinylated antibodies. A Foxp3 fixation kit (eBioscience) was used in conjunction with anti‐Foxp3 (FJK‐16s, eBioscience) or the BD Cytofix/Cytoperm Kit (BD Biosciences) to preserve the GFP signal. Data were acquired using a BD LSR Fortessa and FACSDiva 2.6 software and analysed using FloJo software (Tree Star).

### Preparation and purification of thymic epithelial cells

Thymus tissue was enzymatically digested with Collagenase/Dispase (2.5 mg/ml; Roche) and DNase 1 (40 mg/ml; Roche). After digestion, CD45^+^ thymocytes were depleted using anti‐CD45 microbeads and LS columns (Miltenyi Biotec). Antibodies against the following were used to sort TEC populations: CD45 (30‐F11, eBioscience), EpCAM1 (G8.8, eBioscience), I‐A^b^ (AF6‐120.1, BD Bioscience), Ly51 (6C3, Biolegend), CD80 (16‐10A1, Biolegend). Reagents were conjugated to Pacific Blue, BV 421, BV605, PE, PerCP–eFluor 710, allophycocyanin–eFluor 780. Streptavidin PE‐Cy7 was used to detect biotinylated antibodies. Sorting was performed using a FACS Aria Fusion 1 sorter (BD) with a purity typically >98%.

### qPCR

The following FACS‐sorted TEC populations were analysed by qPCR: cTEC: CD45^−^EpCAM1^+^Ly51^+^; mTEC^lo^: CD45^−^EpCAM1^+^Ly51^−^MHCII^lo^CD80^lo^; mTEC^hi^: CD45^−^EpCAM1^+^Ly51^−^MHCII^hi^CD80^hi^. mRNA isolation, cDNA synthesis and qPCR were performed exactly as described 29. Primer sequences are as follows:

Actb (NM_007393)
QuantiTect Mm_Actb_1_SG primer assay (Qiagen QT00095242)
Ccl20 (NM_016960)
Forward primer 5′‐ACTGTTGCCTCTCGTACATACA‐3′;
Reverse primer 5′‐GAGGAGGTTCACAGCCCTTTT‐3′

### Thymus transplantation

Freshly isolated embryonic day (E) 18 thymus lobes were used as thymus transplants. To assess the role of Aire in thymus recirculation, thymus lobes from WT CD45.1^+^ mice were transplanted under the kidney capsule 52 of congenically marked CD45.2 WT or *Aire^−/−^* mice. To assess the role of CCR6, CD45.2^+^ WT or *Ccr6^−/−^* thymus lobes were transplanted into WT CD45.1^+^ adult mice. After 5 weeks, host thymus and spleen tissues were harvested and graft derived T‐cell subsets identified by expression of CD45.1 or CD45.2, as appropriate.

## Conflict of interests

The authors declare no commercial or financial conflict of interest.

**Abbreviations**
cTEC: cortical thymic epithelial cells
DP: Double Positive
mTEC: medullary thymic epithelial cells
SP: Single Positive

## Supporting information

Peer review correspondence

Supporting Information Figure 1
