# Supplementary material for: Aire controls the recirculation of murine Foxp3+ regulatory T‐cells back to the thymus
Source: Eur J Immunol. 2018 Jan 29;48(5):844–54. doi: 10.1002/eji.201747375 (PMC6001551; doi:10.1002/eji.201747375)
Supplement: Supplementary file 2 — Supporting Information Figure 1 [file EJI-48-844-s002.pdf]

European Journal of Immunology

Supporting Information for DOI 10.1002/eji.201747375

Jennifer E. Cowan, Song Baik, Nicholas I. McCarthy, Sonia M. Parnell, Andrea J. White, William E. Jenkinson and Graham Anderson

Aire controls the recirculation of murine Foxp3^{+} regulatory T-cells back to the thymus

© 2017 The Authors. *European Journal of Immunology* published by WILEY-VCH Verlag GmbH & Co. KGaA, Weinheim.

Cowan et al Supp Figure 1

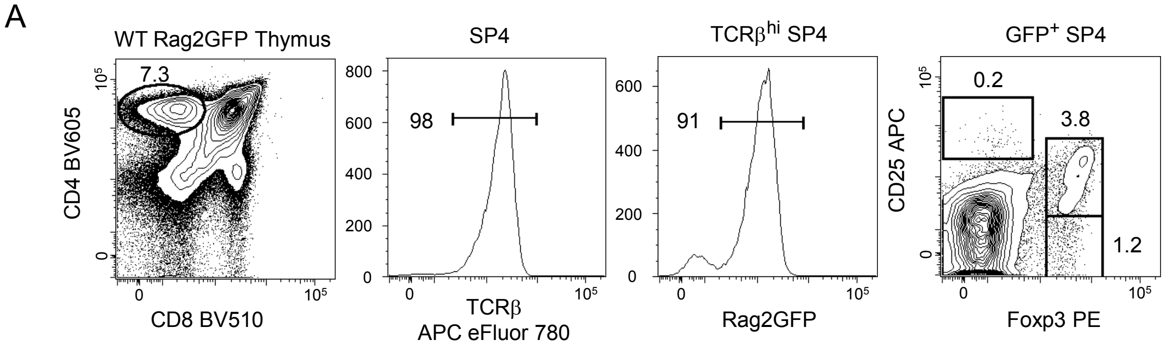

Supplementary Figure Legend

Supplementary Figure 1. Gating Strategy To Identify Foxp3$^+$ T-Reg And Their Precursors In Thymus.

Adult thymocytes stained with anti-CD4 and anti-CD8 antibodies were electronically gated to identify mature CD4$^+$CD8$^-$ thymocytes. CD4$^+$CD8$^-$ cells expressing TCRβ and Rag2GFP were then further gated as indicated, and analysed for their expression of Foxp3 and CD25. Gates on Foxp3/CD25 analysis show Foxp3$^+$CD25$^+$ cells T-Reg, while Foxp3$^+$CD25$^-$ and Foxp3$^-$CD25$^+$ cells represent T-Reg precursors.
