# Supplementary material for: Aire controls the recirculation of murine Foxp3+ regulatory T‐cells back to the thymus
Source: Eur J Immunol. 2018 Jan 29;48(5):844–54. doi: 10.1002/eji.201747375 (PMC6001551; doi:10.1002/eji.201747375)
Supplement: Supplementary file 1 — Peer review correspondence [file EJI-48-844-s001.pdf]

European Journal of Immunology

Aire controls the recirculation of murine Foxp3^{+} regulatory T-cells back to the thymus

Jennifer E. Cowan, Song Baik, Nicholas I. McCarthy, Sonia M. Parnell, Andrea J. White, William E. Jenkinson and Graham Anderson

Correspondence: Dr. Graham Anderson, Institute for Biomedical Research, Medical School, University of Birmingham, Edgbaston, Birmingham, United Kingdom, B15 2TT.

Review Timeline:	Submission date:	16-Oct-2017
	First Editorial decision:	21-Nov-2017
	Revision received:	07-Dec-2017
	Accepted:	20-Dec-2017

Handling Executive Committee member: Prof. Shimon Sakaguchi

Please note that the correspondence below does not include the standard editorial instructions regarding preparation and submission of revised manuscripts, only the scientific revisions requested and addressed.

First Editorial Decision
21-Nov-2017

Dear Dr. Anderson,

We are sorry for the delay in the peer review of your manuscript ID eji.201747375 entitled "Aire Controls The Recirculation Of Foxp3+ Regulatory T-cells Back To The Thymus", which you submitted to the European Journal of Immunology. There was a delay in receiving one of the reports. The comments of the referees are included at the bottom of this letter.

Although the referees have recommended publication, some revisions to your manuscript have been requested. Therefore, I invite you to respond to the comments of the referees and revise your manuscript accordingly.

You should also pay close attention to the editorial comments included below. **In particular, please edit your figure legends to follow Journal standards as outlined in the editorial comments. In more detail please

clarify the number of mice as requested and provide the gating strategy and axis display (flurochromes) for flow cytometry data. Failure to do this will result in delays in the re-review process.**

If the revision of the paper is expected to take more than three months, please inform the editorial office. Revisions taking longer than six months may be assessed by new referee(s) to ensure the relevance and timeliness of the data.

Once again, thank you for submitting your manuscript to European Journal of Immunology. We look forward to receiving your revision.

Yours sincerely,
Nadja Bakocevic

on behalf of
Prof. Shimon Sakaguchi

Dr. Nadja Bakocevic
Editorial Office
European Journal of Immunology
e-mail: ejied@wiley.com

www.eji-journal.eu

****

Reviewer: 1

Comments to the Author
Very nice study that convincingly demonstrates a role for Aire in the recirculation of peripheral Tregs into the thymus. I only have two very, very minor points:
1) On several occasions, the authors mention the use of 'lymphoid' thymic lobes for transplantation?
2) The authors may want to consider rephrasing the header of the last paragraph of the results section (Aire-independent...). This statement is certainly not wrong, but as they do not identify or further specify those 'Aire-independent mechanisms', it might be more straight forward to just state 'Aire is not required for conventional CD4+ thymocyte development' (line 39, page 9)

European Journal of Immunology

Reviewer: 2

Comments to the Author

Cowan et al report on Aire role in recirculation of FoxP3+ regulatory T cells to the thymus. This is another paper on Treg recirculation into the thymus by the same team. The experiments are carefully performed and the paper is well written, and there are no major concerns with this. However, the authors should comment on the possibility of Aire role in long-term retention of Tregs in the thymus (which may contribute to the effect seen in Figure 1D); long-term retention seems to have a larger role than recirculation in establishing the pool of long-lived Tregs (see Yang et al. EJI 44:2712).

First Revision – authors' response

07-Dec-2017

Many thanks for providing us with reviewers comments on our manuscript "**Aire Controls The Recirculation of Foxp3^{+} Regulatory T-cells Back To The Thymus**". We were very pleased to see that both reviewers found our work to be of interest, and acceptable subject to minor revisions. Our changes are summarized below.

Reviewer 1.

- On several occasions, the authors mention the use of 'lymphoid' thymic lobes for transplantation?

Response: We now clarify our use of 'lymphoid' thymus lobes for transplantation on page 6.

- The authors may want to consider rephrasing the header of the last paragraph of the results section (Aire-independent...). This statement is certainly not wrong, but as they do not identify or further specify those 'Aire-independent mechanisms', it might be more straight forward to just state 'Aire is not required for conventional CD4+ thymocyte development' (line 39, page 9)

Response: As suggested, we have now replaced the header of the last paragraph of the results section.

Reviewer 2.

Cowan et al report on Aire role in recirculation of FoxP3+ regulatory T cells to the thymus. This is another paper on Treg recirculation into the thymus by the same team. The experiments are carefully performed and the paper is well written, and there are no major concerns with this. However, the authors should comment on the possibility of Aire role in long-term retention of Tregs in the thymus (which may contribute

to the effect seen in Figure 1D); long-term retention seems to have a larger role than recirculation in establishing the pool of long-lived Tregs (see Yang et al. EJI 44:2712).

Response: We now discuss the possible contributions of recirculation and retention of Foxp3$^+$ T-Reg in the thymus as requested on page 12, and also cite the publication suggested by the reviewer (now reference 42).

In addition, we have also revised our manuscript following editorial comments. Specifically, we:

1. Provide a full gating strategy used to identify Foxp3$^+$ T-Reg and their precursors (Supplementary Figure 1).
2. Clarify the numbers of experiments and animals used in the figure legends.
3. Annotate the flow cytometry plots in figures to indicate scale and the fluorochromes used.
4. Revise the title as suggested.

We hope that these revisions mean that our manuscript is now acceptable for publication in 'European Journal of Immunology'.

<u>Second Editorial Decision</u>

<u>18-Dec-2017</u>

Dear Dr. Anderson,

It is a pleasure to provisionally accept your manuscript entitled "Aire Controls The Recirculation Of Murine Foxp3+ Regulatory T-cells Back To The Thymus" for publication in the European Journal of Immunology. For final acceptance, please follow the instructions below and return the requested items as soon as possible as we cannot process your manuscript further until all items listed below are dealt with.

Please note that EJI articles are now published online a few days after final acceptance (see Accepted Articles: http://onlinelibrary.wiley.com/journal/10.1002/(ISSN)1521-4141/accepted). The files used for the Accepted Articles are the final files and information supplied by you in Manuscript Central. You should therefore check that all the information (including author names) is correct as changes will NOT be permitted until the proofs stage.

We look forward to hearing from you and thank you for submitting your manuscript to the European Journal of Immunology.

Yours sincerely,

Eloho Etemire

on behalf of

Prof. Shimon Sakaguchi

Dr. Eloho Etemire
Editorial Office
European Journal of Immunology
e-mail: ejied@wiley.com
www.eji-journal.eu
